# The Effect of Development in Respiratory Sensory Gating Measured by Electrocortical Activations

**DOI:** 10.1155/2015/389142

**Published:** 2015-06-02

**Authors:** Pei-Ying S. Chan, Chia-Hsiung Cheng, Andreas von Leupoldt

**Affiliations:** ^1^Department of Occupational Therapy, College of Medicine, Chang Gung University, No. 259, Wen-Hwa 1st Road, Taoyuan 333, Taiwan; ^2^Healthy Ageing Research Center, Chang Gung University, No. 259, Wen-Hwa 1st Road, Taoyuan 333, Taiwan; ^3^Research Group on Health Psychology, University of Leuven, Tiensestraat 102, 3000 Leuven, Belgium; ^4^Department of Systems Neuroscience, University Medical Center Hamburg-Eppendorf, Germany

## Abstract

The perception of respiratory sensations can be of significant importance to individuals for survival and greatly impact quality of life. Respiratory sensory gating, similar to somatosensory gating with exteroceptive stimuli, is indicative of brain cortices filtering out repetitive respiratory stimuli and has been investigated in adults with and without diseases. Respiratory gating can be tested with the respiratory-related evoked potential (RREP) method in the electroencephalogram with a paired inspiratory occlusion paradigm. Here, the RREP N1 component elicited by the second stimulus (S2) shows reduced amplitudes compared to the RREP N1 component elicited by the first stimulus (S1). However, little is known regarding the effect of development on respiratory sensory gating. The present study examined respiratory sensory gating in 22 typically developed school-aged children and 22 healthy adults. Paired inspiratory occlusions of 150-ms each with an inter-stimulus-interval of 500-ms were delivered randomly every 2–4 breaths during recording. The main results showed a significantly larger RREP N1 S2/S1 ratio in the children group than in the adult group. In addition, children compared to adults demonstrated significantly smaller N1 peak amplitudes in response to S1. Our results suggest that school-aged children, compared to adults, display reduced respiratory sensory gating.

## 1. Introduction

Respiration is a fundamental vital function in humans. The perception of respiratory sensations can be of significant importance in individuals for survival and greatly impact quality of life, especially in patients with respiratory diseases. However, respiration is usually not sensed unless ventilatory pattern changes or is attended to [1]. Methods for measuring respiratory perception include not only subjective measurements such as self-reporting questionnaires [2–4], but also objective measurements. For example, cortical neuronal activations elicited by inspiratory or expiratory loads can be measured by the respiratory-related evoked potential (RREP) in the electroencephalogram (EEG) [5, 6].

The RREP method has been applied to investigate the cortical processing of respiratory sensations in healthy adults [7, 8], individuals with anxiety disorders [9], asthmatic disease [10, 11], obstructive sleep apnea syndrome [12–14], and central hypoventilation [15]. Some of the aforementioned studies used single-obstruction (odd-ball) paradigms with the RREP method, that is, obstructing once during single inspiration, to explain neural plasticity in the higher brain centers. For example, Davenport et al. (2000) indicated that cortical neural plasticity has been suggested by the absence of RREP component peaks (i.e., no evoked potentials are elicited) in a subgroup of children with life-threatening asthma, where the P1 component was not observed.

The paired-obstruction RREP paradigm (i.e., obstructing twice within single inspiration) was developed to investigate mechanisms of overperception in respiratory sensation. The ability of the higher cortices to process repetitive respiratory sensory stimuli was defined as “respiratory sensory gating” function, which is similar to sensory gating functions tested with auditory and somatosensory stimuli [16–19]. Respiratory sensory gating can be tested by applying paired inspiratory obstructions of 150 milliseconds (ms) each with an inter-stimulus-interval (ISI) of 500 ms [20]. In healthy adults, the RREP elicited by the 2nd stimulus (S2) is smaller in amplitudes than that elicited by the first stimulus (S1), resulting in a N1 component peak amplitude S2/S1 ratio of usually less than 0.5. The RREP S2/S1 ratio is an index of the amount of cortical neural information filtered “in” regarding the important first stimulus and the amount filtered “out” regarding the unwanted or redundant second stimulus and has been investigated in healthy adults [21] and in adults with anxiety disorders [22].

However, these previous RREP studies with paired stimulus designs focused on adult populations. Development could be a potential factor modulating the respiratory sensory gating function, especially when some cortical areas are still not fully mature in preadolescence [23, 24]. In the auditory gating literature, the effect of development on auditory sensory gating has been widely investigated [18, 25–29]. It has been demonstrated that healthy school-aged children demonstrate less auditory P50 peak gating ability compared to healthy adults [18, 26]. Brinkman and Stauder's (2007) study also suggests that age is negatively correlated with P50 S2/S1 ratios. In contrast, respiratory sensory gating has not been tested in children. Understanding respiratory sensory gating function in children is important and can serve as basis for future investigation of neural mechanisms of symptom overperception in childhood diseases such as pediatric asthma. Therefore, the purpose of this study was to investigate respiratory neural gating with the paired RREP paradigm in school-aged children. It was hypothesized that the RREP gating would be reduced in young children as indicated by larger RREP N1 S2/S1 ratios when compared to adults.

## 2. Materials and Methods

### 2.1. Participants

Twenty-seven typically developed healthy children aged between 6 and 12 years and 22 healthy adults aged between 18 and 45 years were recruited for this study. Based on self-report, all participants were nonsmokers and free of any history of respiratory, cardiovascular, or neurological diseases. All participants were instructed to have a sound sleep before the day of the experiment and to avoid vigorous exercise or intake of neural stimulants such as caffeinated drinks before the experiment. The protocol of this study was approved by the Institutional Review Board of the Chang Gung Medical Foundation.

### 2.2. Experimental Procedure

#### 2.2.1. Participants

The adult participants signed their informed consent form. Children of at least 7 years and their legal guardian signed the informed consent, while the children younger than 7 years had their legal guardians signing the informed consent for them. All participants were provided with explanations about the study protocol. After completing the informed consent, the participants underwent a pulmonary function test (PFT) with a spirometer (Cardinal Health Inc., Dublin, OH, USA) in order to ensure adequate lung function. The PFT was conducted according to the guidelines of the American Thoracic Society and European Respiratory Society [30].

#### 2.2.2. Respiratory Apparatus

During the experiment, the participants sat comfortably in an armed chair and wore an electrode cap while breathing through a mouthpiece with a nose clip in position. The mouthpiece was connected to a two-way nonrebreathing valve (Hans Rudolph Inc., Kansas City, USA). The participant's mouth pressure was monitored and recorded from the center of the nonrebreathing valve through a differential pressure transducer connecting to the pneumotachograph amplifier (1110 series, Hans Rudolph Inc., Kansas City, USA). The rest of the apparatus (including the pneumotachograph amplifier) was screened from the participant in the adjacent room. The amplifier was connected to a PowerLab signal recording unit (ADInstruments Inc., Bella Vista, Australia). The setup of the respiratory apparatus has previously been described [20]. The inspiratory port of the nonrebreathing valve was connected to a customized occlusion valve (Hans Rudolph Inc., Kansas City, USA). The two ends of a solenoid, controlled by a trigger box, were connected to the occlusion valve and an air pressure tank via pressure tubing. The closure of the occlusion valve was manually controlled by the experimenter with a trigger box.

#### 2.2.3. The Paired RREP Method

For the details of the RREP method, refer to the previous methodology paper [31]. Briefly, while breathing through the breathing circuit, the participant wore a 40-channel electrode cap (referenced to bilateral mastoids) connecting to an EEG system (NuAmps, Compumedics Neuroscan Inc., Charlotte, NC, USA). The impedance was set below 5 kΩ for every electrode. The EEG signal was sampled at 1 kHz and filtered from DC to 50 Hz. For the experiment, at least 100 paired inspiratory occlusions (150 ms each) with 500-ms ISI were provided randomly every 2 to 4 breaths. The paired stimuli were manually presented at the onset of inspiration by the experimenter triggering the occlusion valve closure via the trigger box. Parallel markers from the trigger box were sent to the Neuroscan recording software (Neuroscan 4.5, Compumedics Neuroscan Inc., Charlotte, NC, USA). During recordings, participants were watching a video (with sound) on a screen in order to be distracted from the stimuli.

### 2.3. Data Analyses

Offline analyses were conducted separately for the S1 and S2 RREP. The onset of mouth pressure change was used as the onset of inspiratory occlusion (LabChart V7, ADInstruments Inc., Bella Vista, Australia). The EEG segments were extracted from 200-ms before till 1000-ms after the stimulus. The signals were baseline corrected according to the initial 200 ms and then again corrected for ocular movement with a built-in algorithm in the analysis software (BrainVision Analyzer 2, Brain Products GmbH, Gilching, Germany). The signal was then bandpass filtered from 1 to 30 Hz. Those signals larger than 100 *μ*V for the 4 eye electrodes and larger than 60 *μ*V for all other electrodes were identified as artifacts and were deleted from the data before averaging.

The RREP Nf, P1, and N1 peaks were identified with latencies and amplitudes calculated separately for S1 and S2. The Nf peak was identified at the frontal F3 and F4 electrodes approximately 25 to 50 ms after the stimulus, P1 peak at the CP3 and CP4 electrodes 50 to 85 ms after the stimulus, and N1 peak at the vertex Cz electrode 85 to 130 ms after the stimulus. The peak amplitudes were determined and the S2/S1 ratios for each peak were calculated. Separate one-way analyses of variance (ANOVA) were performed to test for group differences in age, lung function measures, peak latencies, S2/S1 ratios, and amplitudes. The significance level was set at *p* < 0.05.

## 3. Results

Data of 5 children were excluded from the analysis due to excessive noise in the EEG signals, which left the study with 22 children (12 females and 10 males; 8.6 ± 1.8 years) and 22 healthy adults (10 females and 12 males; 30.8 ± 9.1 years) for final analyses. The demographic and the pulmonary function data of the two groups of participants are shown in Table 1. There was no statistical difference regarding the percentage of predicted values for the forced vital capacity (FVC), for the forced expiratory volume in 1 second (FEV1), and for the ratio FEV1/FVC between children and adults.  Table 2 shows the latencies of Nf, P1, and N1 peaks for the S1 and S2 RREP. There was a statistical difference in S1 Nf and S2 N1 peak latencies between the two groups (*p* = 0.01 and 0.03, resp.) indicating shorter latencies in the children. There was also a trend for shorter S2 Nf and S1 N1 latencies for the children compared to the adults (*p* = 0.05 and 0.06, resp.).

**Figure 1:**
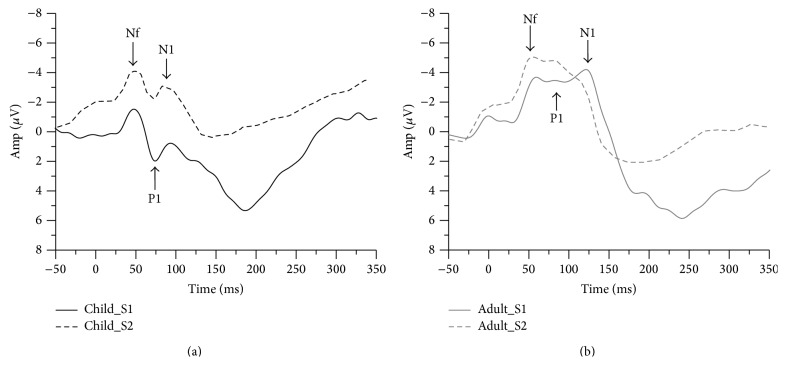
Grand average waveform from the FCz electrode. (a) The black solid and dotted lines represent the averaged S1 and S2 waveforms, respectively, of the children (*N* = 22); (b) the grey solid and dotted lines represent the averaged S1 and S2 waveforms, respectively, of the healthy adults (*N* = 22). Amp: amplitude.

**Figure 2:**
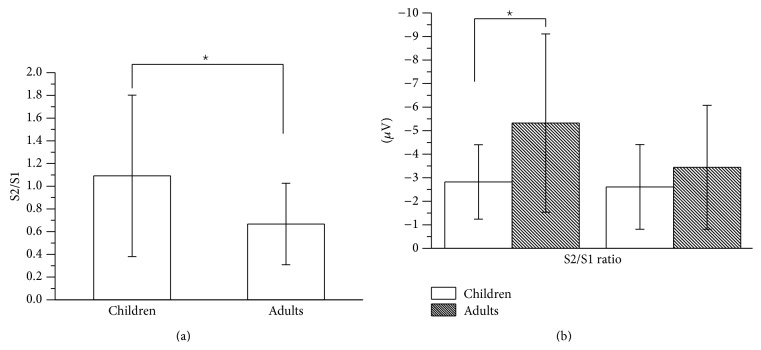
Group averaged RREP N1 peak (a) S2/S1 ratio at the Cz electrode for the children group and the adult group; (b) S1 and S2 amplitudes at the Cz electrode for the two groups. The asterisk ∗ indicates a significant difference between the two groups. Error bars represent the standard deviation.

Figure 1 shows the grand averaged S1 and S2 RREP waveforms of the children group (a) and the adult group (b). One-way ANOVA results showed that the children group demonstrated a significantly larger N1 S2/S1 ratio compared to the adult group (Cz: 1.09 ± 0.71 and 0.67 ± 0.36, resp., *p* = 0.02). The children group also showed a larger Nf S2/S1 ratio compared to the adult group (F3: 1.43 ± 0.75 and 0.97 ± 0.42, resp., *p* = 0.02; F4: 1.49 ± 0.75 and 0.97 ± 0.8, resp., *p* = 0.05). Further analyses on S1 and S2 revealed that the N1 S1 amplitudes for the children group were smaller than for the adult group (Cz: −2.82 ± 1.58 *μ*V and −5.32 ± 3.79 *μ*V, resp., *p* = 0.01), but not for the N1 S2 amplitudes (Cz: −2.61 ± 1.8 *μ*V and −3.44 ± 2.63 *μ*V, resp., *p* = 0.22).  Figure 2 shows a bar graph for the N1 (Cz electrode) S2/S1 ratios of the two groups (a) and a bar graph for the N1 amplitudes for S1 and S2 RREP of the two groups (b). In addition, the Nf S1 amplitudes for the children group were smaller than for the adult group. The S1 and S2 amplitudes and S2/S1 ratios for the Nf, P1, and N1 peaks in the two groups are listed in Table 3.

**Figure 3:**
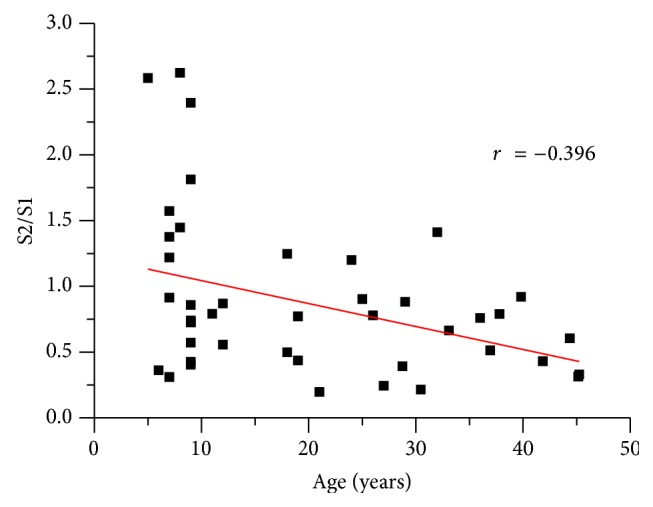
Scatter plot for the correlation of N1 S2/S1 ratio and age for all participants (*N* = 44).

Two-tailed Pearson correlation analyses across all participants revealed that age was significantly correlated with the N1 S2/S1 ratio (*r* = −0.396, *p* = 0.008) and with N1 S1 amplitudes (*r* = −0.353, *p* = 0.019), but not with N1 S2 amplitudes (*r* = −0.06, *p* = 0.697). Age was also significantly correlated with N1 S1 latency (*r* = 0.346, *p* = 0.021). A scatter plot for the age and N1 S2/S1 ratios across all participants is shown in Figure 3.

In addition, the analyses revealed that age was significantly correlated with the Nf peak S2/S1 ratio (F3: *r* = −0.413, *p* = 0.007) and with the Nf S1 amplitude (F3: *r* = −0.395, *p* = 0.01), but not with Nf S2 amplitude (F3: *r* = 0.14, *p* = 0.37). Finally, age was also significantly correlated with Nf S1 latency (*r* = 0.563, *p* < 0.001) and S2 latency (*r* = 0.427, *p* = 0.004).

## 4. Discussion

This experiment has demonstrated that the paired inspiratory occlusion RREP paradigm can be used for measuring respiratory sensory gating function in school-aged children. The significance of the present results lies in the finding that healthy children compared to adults showed reduced respiratory sensory gating as represented by a higher N1 peak S2/S1 ratio. The findings suggest that school-aged children are not as effective as adults in their cortical filtering of repeated respiratory stimuli, which might contribute to differences in perceiving respiratory sensations.

The result of an increased N1 gating ratio in children in the present study is similar to some previous studies on auditory sensory gating [18, 25, 26]. For example, Freedman et al. (1987) tested typically developed children from 18 months to 19 years and found that children compared to adults have reduced gating represented by a larger auditory P50 S2/S1 ratio. They also noted that children from 1 to 8 years exhibited a wide range of P50 S2/S1 ratios. Davies et al. (2009) further suggested that children from 5 to 12 years do not show a mature auditory neural gating mechanism as reflected by increased S2/S1 ratios for auditory ERP components P50 and N100.

In order to understand the factors contributing to the difference in respiratory sensory gating between typically developed children and adults, we conducted further analyses comparing the S1 amplitudes and S2 amplitudes between the two groups. Our results showed that the premature gating in children was due to smaller N1 S1 amplitudes in the children group compared to the adults, which was mirrored by a correlation between age and the N1 amplitude for the S1 RREP, but not for the S2 RREP. This is consistent with the results of Davies et al. (2009) who similarly found a larger auditory P50 S2/S1 ratio in children due to smaller S1 amplitudes compared to the adults. Brinkman and Stauder (2007) reported comparable findings for typically developed young children between 5 and 7 years with auditory stimuli. Together with these findings from the auditory domain, our present results suggest that a less effective respiratory sensory gating function in children is not primarily due to a deficiency in the response to the second stimulus (S2) but rather related to a smaller response to the initial stimulus (S1). This indicates that maturation in respiratory sensory gating may be more of a function of developing enhanced responses to the initial respiratory stimulus in typically developed individuals, which is also supported by our finding of a significant correlation between N1 amplitudes and age.

In addition, the RREP Nf peak S2/S1 ratio and the Nf S1 amplitudes showed the same pattern as the N1 peak in our children when compared to the adult group. We also found a moderate correlation between age and the Nf S1 amplitude. The Nf peak has its source localized at the frontal F3 and F4 electrodes, which represent supplementary motor area precentral cortical generators [8, 32]. It has been considered that the frontal cortex is involved in auditory P50 suppression mechanisms [24]. Marshall et al. (2004) also suggested that the maturation of sensory gating may be related to the prefrontal executive function and attention [29]. Therefore, it is speculated in the present study that the difference in the RREP N1 and Nf amplitudes between adults and children may reflect, in part, the developmental change in executive capacity of the prefrontal cortex. Future investigation is clearly warranted to study further the relationship between the maturation of respiratory sensory gating and the functions of the prefrontal cortex.

Notably, the human frontal lobes are not considered fully developed until individuals reach their 20s, especially in male individuals. Hence, it may be reasoned that this affected the respiratory gating performance of the youngest individuals in our adult group [33]. However, only 2 males out of the 22 individuals in the adult group were younger than 20 years (18 and 19 years, resp.). Therefore, we only expect a minimal effect on the present results in the adult group. In addition, pubertal status can have an important role in brain development and influences children's performance greatly [33]. Although pubertal status was not systematically assessed at the time of our experiment, only two girls out of the 22 individuals in the children group were over 9 years (11 and 12 years, resp.). Therefore, the effect of pubertal status appears to be minimal in the current study. Nevertheless, age cut-off points and pubertal status should be addressed in future investigations.

An additional finding of the present study was a general trend for shorter latencies of the RREP Nf and N1 peaks in the children group as compared to the adult group. This converges with some previous studies in which RREP peak latencies were investigated either in children or in adolescents, respectively. A comparison across these studies shows that the peak latencies in children [34] were usually shorter than those observed in adults [35, 36]. Interestingly, past research on auditory sensory gating in healthy children and adults showed contrasting results with longer peak latencies in children compared to adults and also age-related decreases in auditory P50 peak latency [18, 37]. These differences might be related to the different sensory modalities. In addition, past research has indicated that the process of axonal myelination lasts until individuals reach approximately 40 years of age, which theoretically suggests that peak latencies would be longer in childhood and get shorter when entering adulthood [33]. However, Gogtay and colleagues also mentioned that regions associated with primary functions including primary sensorimotor functions mature first in the brain. Since respiration is one of the most important and vital bodily functions, it could also be reasoned that respiratory-related pathways mature relatively early in development followed by longer peak latencies in adulthood. Future studies directly comparing auditory and respiratory sensory stimuli in single as well as paired RREP occlusion designs between children and adults are, therefore, necessary in order to clarify these contrasting results.

The main limitation of the present study is the lack of additional age groups, which could have helped to delineate more into detail the developmental trajectories in respiratory gating. According to the past studies on auditory sensation, sensory gating functions can vary widely from young childhood to adolescence [18, 25, 29]. For example, Brinkman and Stauder (2007) found that children from 5 to 7 years have significantly worse auditory gating with an age-related decrease in S1 peak latency as compared to those aged 8 years and above. In our dataset, 10 out of the 22 children aged 9 years displayed a wide range of S2/S1 ratios, whereas the only 3 children that were older than 9 years all displayed S2/S1 ratios under 0.9. However, given the relatively small sample size in our study, specific conclusions regarding the mature age in respiratory sensory gating cannot be drawn and require future studies. Moreover, we cannot exclude potential effects on respiratory sensory gating due to mild allergies. Although all participants in this study were free of diagnosed respiratory diseases, a few participants reported regular experiences of nasal allergies. It is known that children with exteroceptive sensory processing deficits exhibit a different pattern in auditory sensory gating [26]. Thus, it is possible that individuals with interoceptive (i.e., respiratory or allergy related) sensory deficits would show different patterns of respiratory sensory gating.

In summary, the present study suggests that typically developed school-aged children show reduced respiratory sensory gating evidenced by reduced RREP N1 peak S2/S1 ratios and smaller S1 peak amplitudes. Whether this pattern of neural processing of respiratory information varies between different age groups in children needs further investigation. Moreover, future research is recommended to determine the factors affecting respiratory sensory gating in children and adolescents.

## Figures and Tables

**Table 1:** Demographic and respiratory variables (mean ± SD). The asterisk *∗* indicates a significant difference between the children group and the adult group (*p* < 0.05).

Variables	Children	Adults
*N*	22	22
Age (y/o)	8.6 ± 1.8	30.8 ± 9.1^*∗*^
Gender (female/male)	12/10	10/12
FEV1 of predicted value (%)	77.86 ± 8.09	81.9 ± 10.37
FVC of predicted value (%)	74.91 ± 7.9	77.24 ± 11.69
FEV1/FVC (%)	92 ± 7.7	91 ± 6.58

FEV1 (L): forced expiratory volume in 1 sec (liter); FVC (L): functional vital capacity (liter).

**Table 2:** Grand averaged S1 and S2 RREP peak latencies (mean ± SD). The asterisk *∗* indicates a significant difference between the children group and the adult group (*p* < 0.05).

RREP latencies (ms)		Children	Adults
Nf peak	S1	47.1 ± 7.4	55 ± 9.9^*∗*^
	S2	47.1 ± 7.6	52.4 ± 10

P1 peak	S1	64.6 ± 12.6	72.1 ± 20
	S2	62.5 ± 10.5	68.2 ± 18.7

N1 peak	S1	102.6 ± 14.5	112.4 ± 26.3
	S2	94.9 ± 13.4	103.9 ± 22.5^*∗*^

**Table 3:** Grand averaged S1 and S2 RREP peak amplitudes (mean ± SD). The asterisk *∗* indicates a significant difference between the children group and the adult group (*p* < 0.05).

RREP amplitudes (*μ*V) and ratios		Children	Adults
Nf peak-F3	S1	−3.15 ± 1.62	−4.24 ± 2^*∗*^
	S2	−4.34 ± 3.03	−3.68 ± 2.44
	S2/S1	1.43 ± 0.75	0.97 ± 0.42^*∗*^

P1 peak-CP3	S1	2.81 ± 1.68	1.95 ± 1.43
	S2	2.45 ± 1.58	1.34 ± 1.07^*∗*^
	S2/S1	1.1 ± 0.86	0.89 ± 0.57

N1 peak-Cz	S1	−2.82 ± 1.58	−5.32 ± 3.79
	S2	−2.61 ± 1.8	−3.44 ± 2.63
	S2/S1	1.09 ± 0.71	0.67 ± 0.36^*∗*^
